# Development and validation of a prediction model for all-cause mortality in maintenance dialysis patients: a multicenter retrospective cohort study

**DOI:** 10.1080/0886022X.2024.2322039

**Published:** 2024-02-28

**Authors:** Jingcan Wu, Xuehong Li, Hong Zhang, Lin Lin, Man Li, Gangyi Chen, Cheng Wang

**Affiliations:** a Department of Nephrology, The Fifth Affiliated Hospital, Sun Yat-sen University, Zhuhai, China; b Guangdong Provincial Key Laboratory of Biomedical Imaging, The Fifth Affiliated Hospital, Sun Yat-sen University, Zhuhai, China; c Department of Nephrology, The First Affiliated Hospital of Guangzhou University of Traditional Chinese Medicine, Guangzhou, China

**Keywords:** Maintenance dialysis, all-cause mortality, pleural effusion, prediction model

## Abstract

**Background:**

The mortality risk varies considerably among individual dialysis patients. This study aimed to develop a user-friendly predictive model for predicting all-cause mortality among dialysis patients.

**Methods:**

Retrospective data regarding dialysis patients were obtained from two hospitals. Patients in training cohort (*N* = 1421) were recruited from the Fifth Affiliated Hospital of Sun Yat-sen University, and patients in external validation cohort (*N* = 429) were recruited from the First Affiliated Hospital of Guangzhou University of Traditional Chinese Medicine. The follow-up endpoint event was all-cause death. Variables were selected by LASSO-Cox regression, and the model was constructed by Cox regression, which was presented in the form of nomogram and web-based tool. The discrimination and accuracy of the prediction model were assessed using *C*-indexes and calibration curves, while the clinical value was assessed by decision curve analysis (DCA).

**Results:**

The best predictors of 1-, 3-, and 5-year all-cause mortality contained nine independent factors, including age, body mass index (BMI), diabetes mellitus (DM), cardiovascular disease (CVD), cancer, urine volume, hemoglobin (HGB), albumin (ALB), and pleural effusion (PE). The 1-, 3-, and 5-year *C*-indexes in the training set (0.840, 0.866, and 0.846, respectively) and validation set (0.746, 0.783, and 0.741, respectively) were consistent with comparable performance. According to the calibration curve, the nomogram predicted survival accurately matched the actual survival rate. The DCA showed the nomogram got more clinical net benefit in both the training and validation sets.

**Conclusions:**

The effective and convenient nomogram may help clinicians quantify the risk of mortality in maintenance dialysis patients.

## Introduction

Despite the fact that more end-stage renal disease (ESRD) patients are living with functional kidney transplants, the number of kidneys that are available for transplantation has not increased in line with the rise in ESRD patients [1]. Currently, dialysis is still the most prevalent treatment modality [2]. In recent years, although there have been considerable improvements in the overall survival rate, the mortality risk for dialysis patients is still between 6.1 and 16.0 times higher than that of the general population [3,4]. Patients receiving dialysis have experienced tremendous burdens. More than 50% of dialysis patients die from cardiovascular disease (CVD), which is considered to be the leading cause of mortality among dialysis patients [5,6]. In addition, uncontrolled hypertension, diabetes mellitus (DM), anemia, and hypoproteinemia are all the high dangerous factors for all-cause mortality of maintenance dialysis patients [7–9]. Worthy of attention is the prognostic value of PE in maintenance dialysis patients. Limited studies have reported that hospitalized patients undergoing maintenance hemodialysis (HD) have a high incidence of pleural effusion (PE). PE (even small amounts of effusion) is an indicator of increased mortality [10,11]. Although we already know some of the risk factors, the mortality risk varies greatly among individual dialysis patients. Therefore, it is a significant issue for the identification and risk stratification of mortality risk among dialysis individuals in clinical practice.

Clinical prediction models have been applied in many diseases such as type 2 diabetes, cancer, and heart failure [12–14]. To date, there have been some published models for predicting mortality in dialysis patients [15–20]. Modifiable risk factors and non-modifiable risk factors are included in these models. However, to our knowledge, these risk factors are not entirely consistent, and several variables are not routinely measured or time-consuming [21,22]. In addition to this, most models are only used for HD or peritoneal dialysis (PD) patients, and models developed may not apply to each other. Most importantly, these models are not well validated. All of these may have led to the fact that more prediction models have been developed in medicine than are actually used in medicine.

Therefore, the objective of this study was to develop and validate a prediction model for predicting long-term survival rates among maintenance dialysis patients based on clinically common and easily accessible data, and visualized in the form of a nomogram. By calculating individual mortality risks, it may be possible for clinicians to assess risks and manage dialysis patients in a more accurate manner. Further, the prognostic value of PE in maintenance dialysis patients was explored.

## Materials and methods

### Collection of data

Hospitalized patients undergoing dialysis were identified and admitted from the two centers between October 2014 and October 2022. First, we excluded repeat hospitalized patients, and excluded participants according to the following exclusion criteria: regular dialysis less than 3 months; only need temporary dialysis; renal transplantation or death within 3 months; HD combined with PD; transform dialysis modality; follow-up less than 3 months; lost follow-up; less than 18 years old.

By browsing the hospital information system, detailed medical histories, clinical characteristics, physical examinations, laboratory data, and chest computed tomography (CT) imaging results were obtained for all patients. We collected laboratory data of the subjects, such as blood routine, serum albumin (ALB), calcium, phosphorus, glycosylated hemoglobin (HbAlc), uric acid, serum creatinine, blood urea nitrogen (BUN), intact parathyroid hormone (iPTH), β_2_-microglobulin, N-terminal pro-brain natriuretic peptide (NT-proBNP), high-sensitivity C-reactive protein (hs-CRP), procalcitonin (PCT), which were measured using a 7180 Biochemistry Auto-analyzer (Hitachi, Tokyo, Japan) in the central laboratory. According to the chest CT imaging results, patients were divided into PE group and non-PE group.

The study protocol was approved by the Ethics Committee of the Fifth Affiliated Hospital of Sun Yat-sen University (Zhuhai, China), which waived the requirement of obtaining written informed consent from all participants. The approval number is: Fifth Affiliated Hospital of Sun Yat-sen University [2022] Lun Zi No. (K151-1).

### Endpoint and follow-up

The endpoint of our study was defined as all-cause mortality from the time of enrollment. Medical records, family members, or the staff of the hospital provided confirmation of the endpoint events. In accordance with the study protocol, follow-up was performed every 3 months via outpatient and/or telephone consultation. In addition, a very important follow-up pathway was through the electronic medical records and registration records of the blood purification center and PD management Center. The deadline for follow-up was 1 October 2022.

### Statistical analysis

Data were tested for normal distribution using the Kolmogorov–Smirnov test. Continuous variables were presented as means ± standard deviations, and significant differences between two groups were determined with Student’s *t*-test. For non-normally distributed data, median and interquartile ranges were used to describe the features, while comparisons of the two sets were performed using a Mann–Whitney *U*-test. Categorical variables were described using counts and percentages, and groups were compared using *χ*^2^ test or Fisher’s exact probability test. We performed multiple imputations for missing values based on five replications.

LASSO-Cox regression was applied to screen out the variables from clinical variables. The optimal prognostic variables were determined by drawing vertical lines at the optimal values given by the minimum criteria and 1se criteria. Then, we applied Cox regression to perform multivariate survival analysis and finally constructed a nomogram. The hazard ratio (HR) and 95% confidence interval (CI) were reported. Time-dependent *C*-indexes at 1-, 3-, and 5-years were generated to assess prognostic accuracy. The calibration curve was applied to determine the observed and predicted nomogram probabilities, while decision curve analysis (DCA) was used to assess the clinical usefulness of the model.

Statistical analyses were performed using SPSS statistics version 25.0 (IBM Corp., Released 2017, IBM SPSS Statistics for Windows, Armonk, NY) and R software version 4.0.3 (R Foundation for Statistical Computing, Vienna, Austria, www.r-project.org). All tests were two-sided. *p* values of less than .05 were considered statistically significant in all analyses.

## Results

### Baseline characteristics of participants

**Figure 1.**
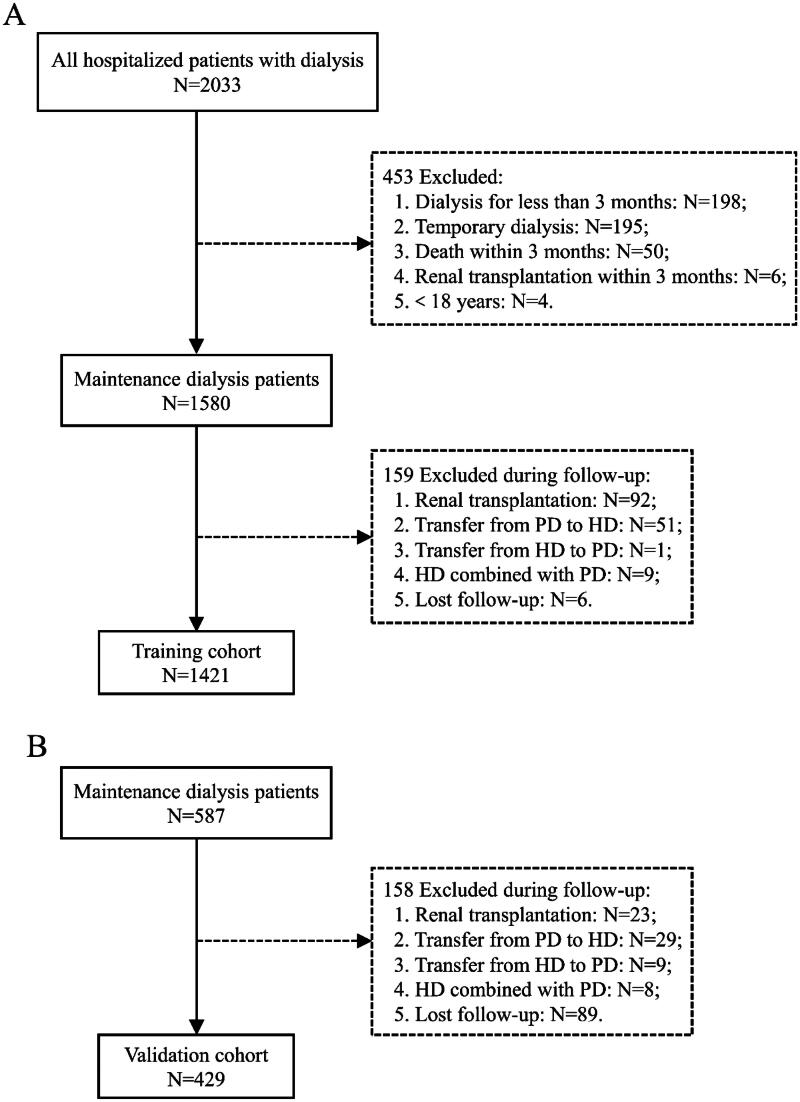
Enrollment and outcomes of the cohorts. PD: peritoneal dialysis; HD: hemodialysis. (A) Training cohort. (B) Validation cohort.

In this study, we initially enrolled 2033 consecutive hospitalized patients with dialysis from the Fifth Affiliated Hospital of Sun Yat-sen University, and 587 participants were identified who were admitted to the First Affiliated Hospital of Guangzhou University of Traditional Chinese Medicine. According to the pre-defined inclusion and exclusion criteria, 1421 participants were ultimately included in the training cohort study, and 429 patients were in the external validation cohort study (Figure 1). A comparison of the two cohorts revealed statistically significant differences in many demographic characteristics, comorbid conditions, symptoms and signs, and laboratory data (*p* < .050), especially in terms of the proportion of HD, the incidence of PE (28.15% in training cohort and 34.27% in validation cohort), DM, CVD and hypertension, the external validation group was significantly higher. A detailed description of the baseline characteristics can be found in Table 1.

**Table 1.** Baseline characteristic of the training and validation set.

Variables	Training set (*N* = 1421)	Validation set (*N* = 429)	*p* Value*
Age (years)	54.46 ± 15.09	57.69 ± 14.47	<.001
Male (*n* (%))	811 (57.07)	239 (55.71)	.618
BMI (kg/m^2^)	22.60 ± 3.68	21.72 ± 3.36	<.001
Clinic-SBP (mmHg)	146.92 ± 25.53	154.95 ± 26.51	<.001
Clinic-DBP (mmHg)	85.56 ± 16.05	88.32 ± 15.25	.002
HR (bpm)	83.58 ± 14.11	83.99 ± 13.49	.595
Dialysis duration (months)	10.00 (4.00, 40.00)	12.00 (5.00, 30.00)	.022
Urine volume (mL)	300.00 (0.00, 800.00)	300.00 (100.00, 800.00)	.001
Dialysis modality (PD/HD)	399/1022	17/412	<.001
PE (*n* (%))	400 (28.15)	147 (34.27)	.015
*Comorbid conditions*			
DM (*n* (%))	419 (29.49)	183 (42.66)	<.001
Hypertension (*n* (%))	1219 (85.78)	402 (93.71)	<.001
CVD (*n* (%))	478 (33.64)	217 (50.58)	<.001
Cancer (*n* (%))	75 (5.28)	24 (5.59)	.799
*Symptoms and signs*			
Chest tightness (*n* (%))	286 (20.13)	136 (31.70)	<.001
Chest pain (*n* (%))	46 (3.24)	13 (3.03)	.831
Expectoration (*n* (%))	192 (13.51)	146 (34.03)	<.001
Fever (*n* (%))	91 (6.40)	22 (5.13)	.334
Legs edema (*n* (%))	410 (28.85)	144 (33.57)	.062
*Laboratory data*			
WBC (×10^9^/L)	6.43 (5.05, 7.97)	7.09 (5.55, 8.79)	.129
HGB (g/L)	98.69 ± 23.43	100.59 ± 25.31	.200
ALB (g/L)	36.62 ± 5.75	38.18 ± 5.82	<.001
Serum calcium (mmol/L)	2.18 ± 0.61	2.22 ± 0.23	.030
Serum phosphate (mmol/L)	1.76 ± 0.61	1.98 ± 0.70	<.001
Serum creatinine (μmol/L)	780.00 (596.50, 990.50)	823.00 (613.50, 1067.00)	.001
BUN (mmol/L)	18.60 (13.72, 24.10)	20.92 (15.36, 27.32)	<.001
Uric acid (mmol/L)	404.26 ± 173.10	402.44 ± 137.33	.842
HbAlc (mg/dl)	5.26 (4.80, 5.90)	6.31 (5.16, 7.60)	<.001
β_2_-microglobulin (mg/L)	27.55 (19.35, 35.58)	29.59 (18.35, 39.63)	.057
iPTH (pmol/L)	28.50 (11.68, 63.05)	22.70 (9.05, 55.39)	.011
NT-proBNP (pg/mL)	9480.00 (2320.00, 33350.00)	2321.00 (750.05, 10605.40)	<.001
hs-CRP (mg/dl)	6.30 (0.30, 31.20)	17.84 (3.76, 53.46)	<.001
PCT (ng/mL)	0.52 (0.17, 2.68)	0.71 (0.19, 4.54)	.008

BMI: body mass index; SBP: systolic blood pressure; DBP: diastolic blood pressure; HR: heart rate; PD: peritoneal dialysis; HD: hemodialysis; PE: pleural effusion; DM: diabetes mellitus; CVD: cardiovascular disease; WBC: white blood cell; HGB: hemoglobin; ALB: albumin; BUN: blood urea nitrogen; HbAlc: glycosylated hemoglobin; iPTH: intact parathyroid hormone; NT-proBNP: N-terminal pro-brain natriuretic peptide; hs-CRP: high-sensitivity C-reactive protein; PCT: procalcitonin.

Cardiovascular disease included congestive heart failure, angina pectoris, and atherosclerotic heart disease. Data are presented as means and standard deviations (SD), numbers (*N*) and percentages (%), or median and quartile ranges.

* *p*, for comparison between training set and validation set.

In the training cohort, the median follow-up period for the study was 28.0 months (range 14.3–47.0 months), and the longest was 89.1 months. A total of 264 patients (18.58%) died during the period of follow-up. Correspondingly, the median follow-up period was 42.4 months (26.4–56.5 months) in the validation cohort. A total of 177 (41.26%) patients experienced endpoint events during the follow-up period.

### LASSO-Cox regression method to select optimal variables

**Figure 2.**
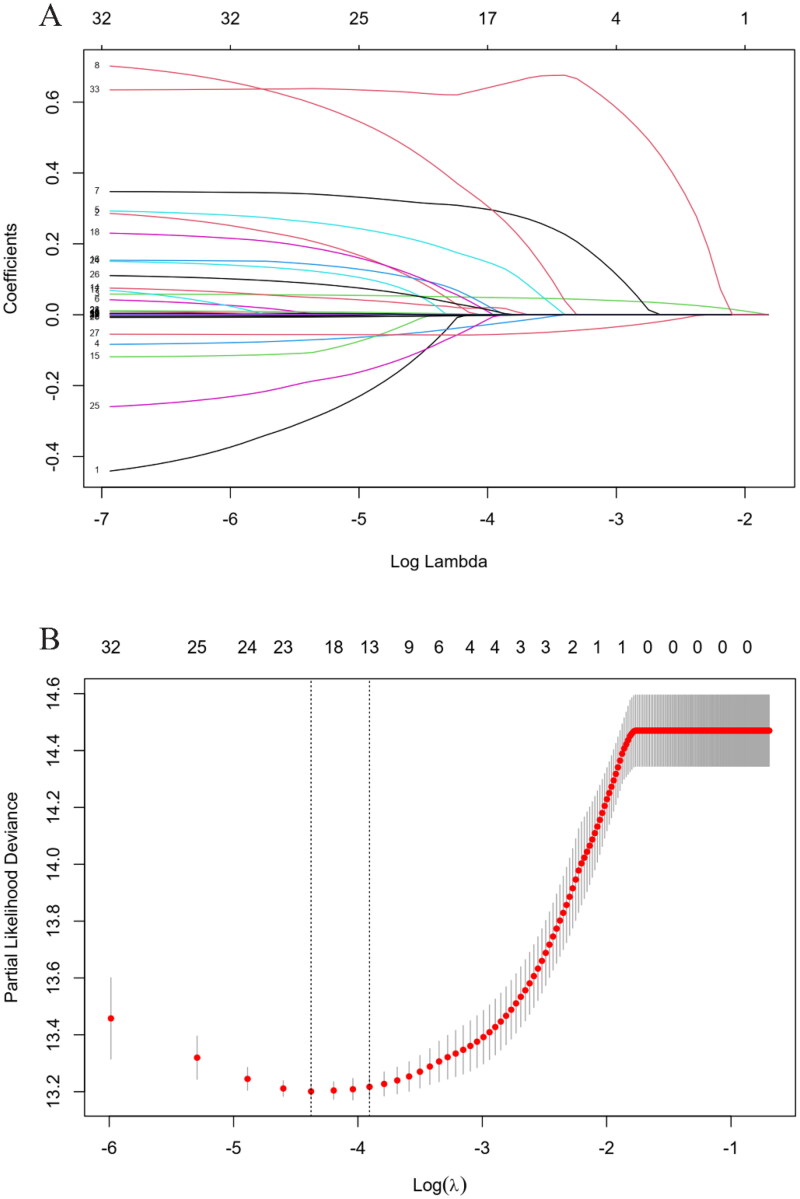
Identification of predictive factors using the LASSO-Cox regression. (A) LASSO model coefficient trendlines of the 33 variables (shown in Table 1) for all-cause mortality. (B) Tuning parameter *λ* selection threefold cross-validation error curve. Vertical lines were drawn at the optimal values given by the minimum criteria and 1es criteria. The parameter *λ* = 0.02 was selected under the 1es criteria, 13 variables of them with non-zero coefficient were selected. LASSO: least absolute shrinkage and selection operator; es: standard error.

LASSO-Cox regression was applied to analyze the correlation between variables and all-cause mortality. With increases in *λ*, the coefficient of variables decreased. When the residual sum of squares was shown to be 1es (*λ* = 0.02) for all-cause mortality, 11 variables with non-zero coefficients were selected for multivariate analysis (Figure 2(A,B)), including age, body mass index (BMI), DM, CVD, cancer, urine volume, chest tightness, expectoration, hemoglobin (HGB), serum ALB, HbAlc, NT-proBNP, and PE.

### Cox regression for all-cause mortality

**Figure 3.**
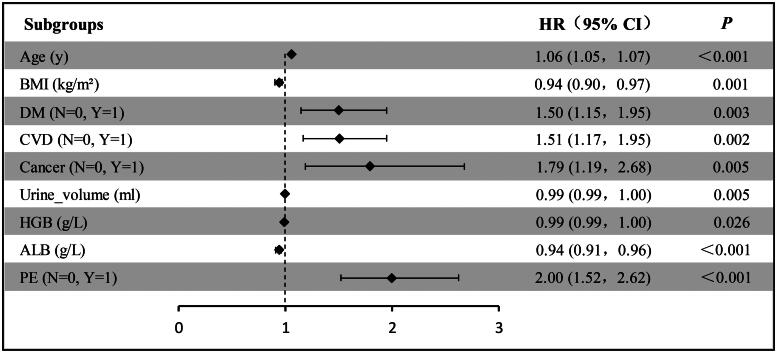
Forest plot with hazard ratio (HR) for the optimal prognostic variables of the final multivariable model in the training set. HR above one indicates that a variable is positively associated with the event probability and negatively with survival time. A horizontal line parallel to the *X*-axis has a logarithmic scale that represents a more precise CI (95% CI).

Multi-variable Cox regression analysis was carried out to further verify the HR and coefficient for each variable reselected by the LASSO-Cox method. As a result, age, BMI, DM, CVD, cancer, urine volume, HGB, ALB, and PE remained independent predictors of all-cause mortality (Figure 3). Eventually, these factors were applied to the nomogram.

### Prediction nomogram of 1-, 3-, and 5-year all-cause mortality

**Figure 4.**
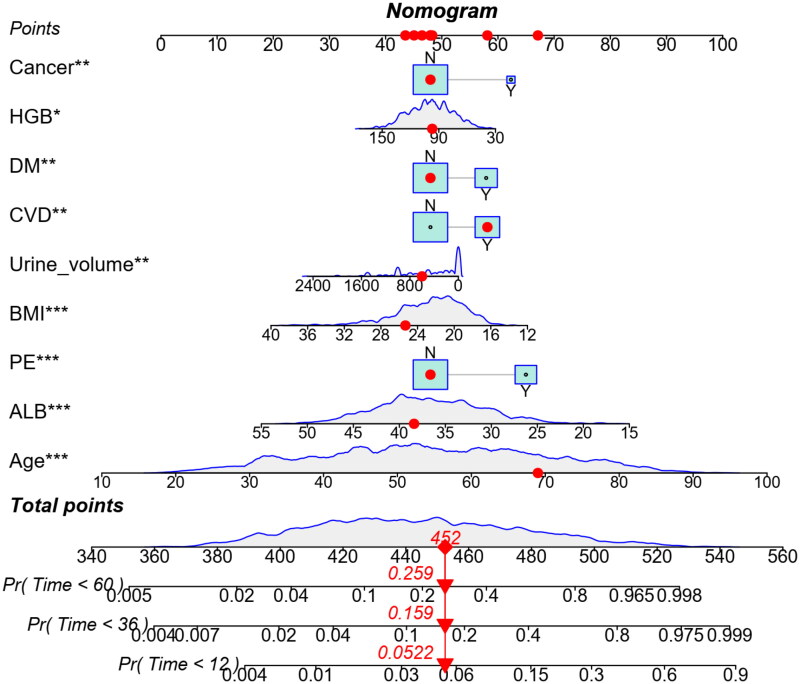
Predictive nomogram for the probability of 1-, 3-, and 5-year all-cause mortality among maintenance dialysis patients. *p < .05; **p < .01; ***p < .001.

Based on multivariate analysis, nine variables were included in the nomogram for predicting 1-, 3-, and 5-year all-cause mortality. The predicted probability can be obtained quickly by adding up the scores for each factor read on the point’s scale of the nomogram. As an example, a 69 years old patient with CVD, BMI 25.33 kg/m^2^, urine volume 600 mL, HGB 97 g/L, ALB 38.40 g/L, without DM, cancer, and PE, the total score will be 452. The result corresponds to 1-, 3-, and 5-year risk of death of 0.052, 0.159, and 0.259, respectively. Correspondingly, the patient’s survival probability at 1-, 3-, and 5-year will be approximately 94.8%, 84.1%, and 74.1%, respectively (Figure 4).

### Web-based dynamic nomogram

A web-based calculator was built with these risk factors (https://dynnom15093191611.shinyapps.io/MY_DN_App/) to facilitate the use of the nomogram for clinicians. We can easily get the survival time (days) and read the output generated by the website.

### Performance of prediction nomogram

**Figure 5.**
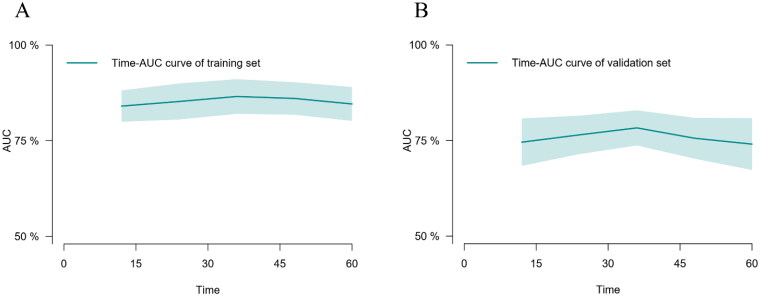
The time–AUC curve analyses between the training set (A) and the validation set (B).

The predictive performance of our model for the prognosis of maintenance dialysis patients was evaluated using time-dependent discrimination analysis. With time-dependent *C*-indices of 0.840, 0.866, and 0.846 at 1, 3, and 5 years in the training set, the corresponding *C*-indices of internal validation were 0.820, 0.822, and 0.808 through 1000 bootstrap replicates, indicating that the nomogram performed well in all-cause mortality prediction. In addition, the accuracy of the nomogram performed well in the external validation set as well, with *C*-indexes of 0.746, 0.783, and 0.741 at 1, 3, and 5 years, respectively. The time–AUC curves of this study show that the predictive power of the nomogram model has remained relatively stable over time, demonstrating that it can be used to predict death over a wide range of time periods in both training set (Figure 5(A)) and validation set (Figure 5(B)).

**Figure 6.**
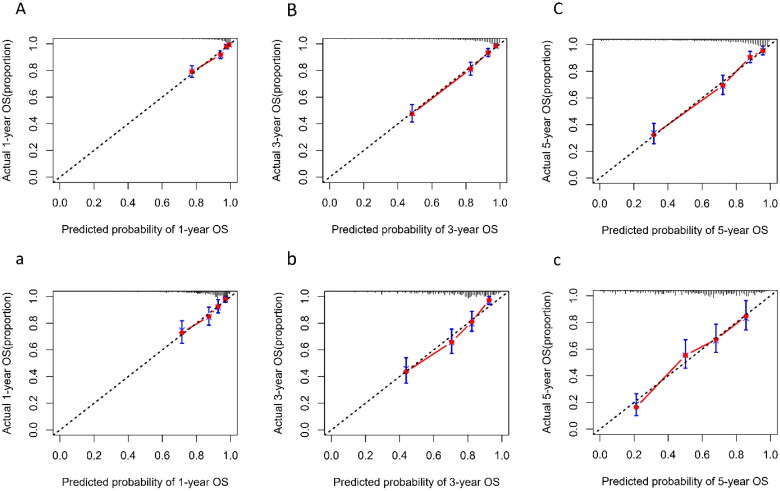
Calibration plot of nomogram predicted probability of 1-year (A), 3-year (B), and 5-year (C) all-cause mortality in training set, and 1-year (a), 3-year (b), and 5-year (c) in validation set. The dashed line represents an ideal evaluation, whereas the red line represents the performance of the nomogram.

The calibration plot indicated that the nomogram was well-calibrated, which meant predicted probabilities close to observed probabilities in both training sets (Figure 6(A–C)) and validation set (Figure 6(a–c)).

**Figure 7.**
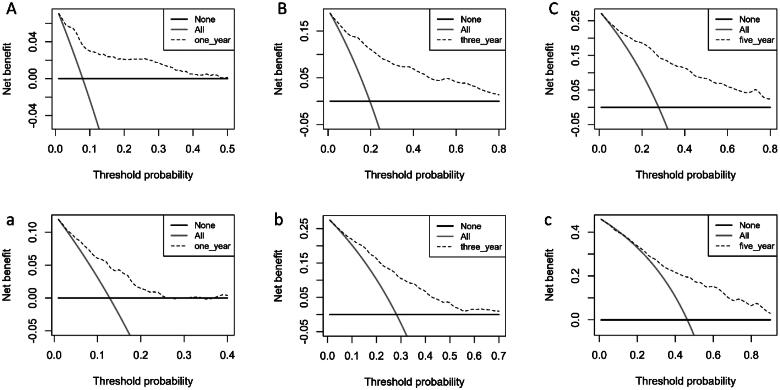
Decision curve evaluates the clinical benefit of the nomogram prediction model for 1-year (A), 3-year (B), and 5-year (C) in training set, and 1-year (a), 3-year (b), and 5-year (c) in validation set. The dashed line represents the nomogram. The black horizontal line represents the net benefit when no patients were considered to exhibit death, while the gray real line represents the net benefit when all patients were considered to exhibit death.

The clinical prediction model was assessed with DCA to determine its net benefit. Using the nomogram developed in this study, the results show a greater net benefit to predict all-cause mortality compared with the treat-all strategy or treat-none strategy, both in the training set (Figure 7(A–C)) and validation set (Figure 7(a–c)).

## Discussion

In the present study, we confirmed that hospitalized patients undergoing maintenance HD have a high incidence of PE, and PE was an indicator of increased mortality. Importantly, we developed a nomogram to predict 1-, 3-, and 5-year survival among maintenance dialysis patients. Multivariate survival analysis showed that age, BMI, DM, CVD, cancer, urine volume, HGB, ALB, and PE were independent predictors for all-cause mortality. At the meantime, the predictive models we have developed have been rigorously externally validated, thereby increasing the clinical applicability of the models.

To the best of our knowledge, predictors that were not routinely collected were used in many studies; these predictors included socioeconomic status, left ventricular ejection fraction, surprise question, peritoneal equilibration test, vascular calcification score, etc. [23–27]. This had led to certain difficulties in the development and widespread clinical application of predictive models. Nevertheless, the predictors in our established prediction model were easily and routinely collected in clinical care, and they were divided into modifiable factors and non-modifiable factors. Therefore, we can achieve timely identification and intervention of these modifiable factors.

It is worth noting that our study also incorporated a new predictor compared to other studies, namely PE. PE is a common clinical presentation in patients undergoing maintenance dialysis [28,29]. The incidence of PE is 28.15% in the training cohort, and the high incidence is notable. Some studies have reported the PE is associated with high mortality in dialysis patients [10,30]. A study on the negative impact of PE on the prognosis of maintenance dialysis patients was conducted by our group and was currently published. The results of the study showed the presence of PE was significantly associated with mortality and was not related to the size of the fluid volume [11]. This is the reason why our study did not conduct a subgroup analysis of PE. The very strong dependency of the association of PE with all-cause mortality may stimulate further clinical and basic science studies.

To the best of our knowledge, a majority of the studies were conducted on HD patients rather than PD patients, and models developed may be not applicable with each other. HD and PD patients showed some distinct differences, as expected. However, although most models were not designed specifically for PD patients, they showed better discrimination for this patient group [31]. A relatively small number of studies were developed in the general dialysis patients (both HD and PD) [32,33], and no further discussion of dialysis modalities was developed. Our study included dialysis modality as a variable to build a predictive model, the results showed no significant difference in the overall prognosis of HD patients compared to PD patients. Previous have shown that PD was preferable to HD among younger patients without DM and with few complications; conversely, HD was more appropriate [34–36]. For the development of easy-to-use predictive models, no further subgroup analysis was performed in our study. Future research on the prognostic differences between HD patients and PD patients could be conducted.

In addition, our study cohort did not exclude patients with cancer. It is well known that cancer history can affect the prognosis of patients; survivors are frequently excluded from cancer clinical trials and observational research [37]. The number of cancer survivors is growing rapidly, but little is known about their treatment and survival needs [38]. Observational studies often provide real-world data for analysis; therefore, the inclusion of patients with prior cancer in observational studies is important to advance evidence-based practice [39]. Based on this, we developed a predictive model by applying a real-world study cohort that did not exclude cancer patients. Additionally, although NT-proBNP was not included in our final prediction model, it has been found that NT-proBNP concentrations are related to left ventricular filling pressures and wall stress, especially when ventricular fibrosis is present [40,41]. In different studies, NT-proBNP threshold levels associated with heart failure varied from 30 to 400 pg/mL [42]. However, in maintenance dialysis patients, the threshold level of NT-proBNP is relatively high. A systematic review and meta-analysis found 37 relatively high-quality studies reported NT-proBNP level relationships with all-cause mortality outcomes in maintenance dialysis patients, and revealed NT-proBNP level elevations at thresholds >1000 up to >20,000 pg/mL were associated with significantly greater risk for all-cause mortality [43]. Future research on this area will be conducted in our group. Beside these predictive factors, age, BMI, DM, CVD, urine volume, HGB, and ALB have been already recognized as independent predictors and have been reported in previous studies [44,45].

According to our results, an external validation set was used to validate the established nomogram, and the performance of the model was good in terms of discrimination and calibration. A noteworthy point was that the *C*-indexes at 1-, 3-, and 5-year survival prediction were above 0.84 in the training set, and above 0.74 in the external validation sets. According to prior research, the models were reported to perform well in terms of discrimination, with *C*-indexes ranging from 0.71 to 0.75 for 1-year time frame, but as the prediction horizon was extended, these models tested for different time frames consistently performed poorly and performed more poorly in external validation [31]. This illustrates that it is critical to perform external validation before implementing clinical trials. In our study, we also performed calibration curves and DCA analyses, and the results all performed better. This means that the performance of our model is better and relatively stable over time.

### Strengths and limitations

In the present study, our analysis has a few strengths. First, this was a multicenter retrospective cohort study, our model had undergone rigorous external validation. The *C*-indexes of our study were all above 0.74, and the performance of the model was good in terms of discrimination and calibration, which means that our nomogram has some general applicability and can improve clinical outcomes to some extent. Second, variables included in the study can be collected readily and routinely in clinical practice. Third, we had a relatively large study cohort with regular follow-up, and we had a very low rate of follow-up loss. Fourth, our prediction model was applicable to both HD and PD patients. Fifth, the LASSO-Cox regression method was used to select optimal variables to avoid multicollinearity between variables.

However, the current study is also subject to certain limitations. First, there was no consideration of DM or CVD comorbidity severity. Second, the current model did not include some factors that have been shown to predict survival risk in dialysis patients, such as troponin T, assessment of ventricular function by echocardiography, extent of vascular calcification, *Kt*/*V* and other indicators to assess dialysis adequacy. Finally, our study also did not consider the impact of medication on patient prognosis, which was due to the fact that our study spanned a relatively long period of time (from October 2014 to October 2022) resulting in a large amount of missing data on patient’s medication status. Therefore, for future research, we need to further improve the performance of the predictive model, and an evaluation of the model in a wider population of dialysis patients is needed.

## Conclusions

We further validated the critical prognostic value of PE for all-cause mortality. A risk prediction tool was also developed and validated for estimating the risk of all-cause mortality in maintenance dialysis patients at 1, 3, and 5 years. Our predictive model includes more objective factors, easier clinical access, and also identifies predictors with reversal potentials. Based on an estimate of each individual’s risk, lifestyle and medical interventions can be made more informed by physicians and patients. Nevertheless, more extensive validation of our model in more central study cohorts is still needed before it can be routinely applied in clinical practice.

## Data Availability

The data underlying this article will be shared on reasonable request to the first author and corresponding author.
